# The role of trachelectomy in cervical cancer

**DOI:** 10.3332/ecancer.2015.506

**Published:** 2015-02-03

**Authors:** MJ Halaska, H Robova, M Pluta, L Rob

**Affiliations:** Department of Obstetrics and Gynaecology, Second Medical Faculty, Charles University, Prague 150 00, Czech Republic

**Keywords:** cervical cancer, chemotherapy, pregnancy outcome, trachelectomy

## Abstract

Cervical cancer is one of the most common cancers in women worldwide. Because it often affects women of childbearing age (19–45 years), fertility-sparing surgery is an important issue. The article reviews current viable fertility-sparing options with a special focus on trachelectomy, including vaginal radical trachelectomy, abdominal radical trachelectomy and simple trachelectomy. Neoadjuvant chemotherapy is also discussed. Finally, the decision to proceed with fertility-sparing treatment should be a patient-driven process.

## Introduction

Cervical cancer is the fourth most common cancer in women worldwide [1]. Striking differences in the incidence of cervical cancer exist between developing and developed countries. In the European Union, 20.9% of women are diagnosed with cervical cancer under the age of 40, which often poses a complication regarding pregnancy plans of women. The average age of women who give birth in Europe is 30.1 years [2]. Because of an increase in coverage of screening programs in Europe, the vast majority of women are diagnosed with early stage cervical cancers (FIGO IA1-IB1), which represent a group of patients considered to be candidates for fertility-preserving procedures.

Reduction of the radicality of surgical procedures has been introduced in numerous malignancies. The most radical steps have been taken in breast cancer surgery, reducing original radical-modified mastectomy with complete axillary lymphadenectomy to a segmentectomy with sentinel lymph node mapping (SLNM). Similar attempts were performed by Professor Daniel Dargent in 1986, including laparoscopic pelvic lymphadenectomy combined with vaginal radical trachelectomy (VRT). Dargent published his experiences in 1994 [3]. An alternative procedure performed abdominally (abdominal radical trachelectomy, ART) was presented by Smith and a collaborative group in 1997 [4].

A randomized controlled comparison study of fertility-preserving techniques is impossible to perform because of the patient’s desire to preserve fertility. Therefore, case-controlled studies have been used to verify oncological results. The safety of VRT has been shown in a Toronto study of 137 patients with a 99% 5-year survival rate [5]. The safety of ART has been reviewed recently in 485 patients from 29 articles, where only 3.8% patients experienced a recurrence and 0.4% died after a median follow-up of 31.6 months [6]. Around 85% of patients were able to retain their fertility.

Several reports have shown that patients with tumours of less than 2 cm in diameter and negative pelvic lymph nodes have minimal risk of parametrium involvement, making a resection of parametrium in any extension unnecessary. Thus, in these cases a simple trachelectomy (ST) procedure combined with pelvic lymphadenectomy is only required [7–11]. In a set of 32 women, no recurrence was diagnosed at median follow-up of 62 months, while 53.1% of women became pregnant after the procedure [12].

Recently, several groups attempted to evaluate fertility preservation by ART in patients with these tumours. An Hungarian group followed 31 patients with cervical cancer IB1 (over 2 cm) and IB2 after ART with 93.5% of 5-year survival rate, which is surprisingly high, and with 9.7% of delivery rate [13]. Another set of 29 patients with tumours of 2–4 cm large from Memorial Sloan-Kettering Cancer Center in New York was published [14]. Due to positive margins or lymph nodes only nine patients (31%) retained their fertility, out of them one patient got pregnant but underwent first trimester termination. Contrary to these results, a Chinese data set of 62 patients with cervical cancer between 2 and 4 cm found fertility preservation possible in 88.7% of patients with no recurrence at 30.2 months of follow-up [15].

The aim of this work is to review current knowledge on the indications for fertility-preserving procedures and significant differences in the techniques used.

## Preoperative workout

Strict indication criteria must be followed to achieve the best oncological results while retaining fertility preservation. One of the most important conditions is the patient’s strong desire to preserve fertility. Based on the current literature, these procedures are oncologically safe in patients with tumours smaller than 2 cm in size with at least 5 mm clear margin after resection and indication of negative pelvic lymph nodes. First, a preoperative biopsy provides basic prognostic information, such as histopathologic type and perineural and lymphovascular involvement. Neuroendocrine and small cell cancer are not considered suitable for fertility-sparing procedures in that they are often associated with a poor prognosis and a high risk of distant metastasis. Cone biopsy is essential for precise diagnosis of clinically undetectable cervical cancer. A description of the cone’s dimensions is extremely important. Colposcopy and clinical examination are another integral part of preoperative assessment, especially in clinically evident disease. In recent years, an integration of effective imaging techniques has been employed. Ultrasonography (US), and particularly magnetic resonance imaging (MRI), can be used to determine the dimensions of the tumour, amount of healthy cervical stroma and a description of the involvement of the parametrium [16]. The US has an advantage because it is an easy to use instrument; however, the examination itself is highly examiner-dependent. Assessment of lymph nodes involves intensive work-up. In patients with early cervical cancer computed tomography (CT), MRI and positron emission tomography (PET) have shown low precision in detecting lymph node metastatic disease [17, 18]. The most precise method to date is SLNM during pelvic lymphadenectomy [17].

Concerning the choice of a procedure for IA1 tumours without lymphovascular space invasion (LVSI), a simple cone biopsy is often executed. Best results are obtained by electrosurgical procedures using a needle (NETZ) or loop (LLETZ) with radiofrequency energy. In IA1 with LVSI tumours, a lymphadenectomy must be combined with cone biopsy [19]. For IA2 tumours, usually VRT with pelvic lymphadenectomy is chosen though some centres also use cone biopsy or ST combined with lymphadenectomy [20]. The most challenging group of tumours is FIGO stage IB1 tumours. For tumours smaller than 2 cm and stromal invasion of less than 10 mm (or half of the stroma), VRT or ART are usually indicated [21]. IB1 tumours larger than 2 cm and IB2 tumours that have a 30–40% risk of lymph node involvement are considered impropriate for fertility-sparing procedures or with a low chance of achieving a fertility preservation [22].

## Trachelectomy surgical techniques

Vaginal radical trachelectomy (VRT) is a modification of the original Schauta-Stockel procedure. VRT should start with laparoscopic pelvic lymphadenectomy (could be combined with detection of SLNs). Eventually, in regions with less-advanced laparoscopic skills, an abdominal approach can be used but the procedure loses its minimal invasivity, which is essential for the future quality of life and excellent cosmetic results. The other part of the procedure consists of a VRT with the resection of the parametria at the level of radical hysterectomy type B. During VRT, the uterine artery is preserved and only the vaginal branch of the artery is transsected. The cervix should be transsected 1 cm above the margin of the tumour while preserving at least 1 cm of cervical stroma caudally from the internal cervical orificium [23]. Adherence to this guideline could be verified by peroperative frozen section examination of the excised cervix but preferably final pathologic examination should be the determining factor [24].

ART was first described in 1932 by Romanian gynaecologist Eugen Aburel but prospectively used as a fertility-sparing procedure by Smith. ART is a modification of the radical abdominal hysterectomy approach, which brings an advantage of minimal special surgical experience except of radical abdominal hysterectomy technique. The first step in the procedure involves pelvic lymphadenectomy. Similarly, the detection of a SLN could be implemented. In the majority of surgical schools, the uterine artery is completely resected though modification with preservation and reanastomosis of the artery has been described [25]. Resection of the parametrium could vary in which resection could be performed according to radical hysterectomy type B or C (with or without nerve-sparing techniques) [26]. The cervix is resected completely and the vagina is sutured directly to the remaining stroma.

ST as a fertility-sparing procedure was first described by our group in 2007 [27]. The procedure employs a two-step management of the patients to ensure the highest oncological safety. In the first step, laparoscopic pelvic lymphadenectomy is used in combination with SLNM. In the case of negative SLNs after final pathological evaluation, ST is performed approximately 1 week later. If positive SLNs are detected, the fertility-preserving procedure is abandoned. ST involves amputation of the cervix combined with the resection of the remaining endocervical channel by loop excision. Outer cervical edges are sutured with vaginal edges to achieve optimal postoperative results. Cervical cerclage is not performed. Other reports have used deep laser conisation with adjuvant chemotherapy [22] or a similar technique as described by the Prague group [28].

A recent survey conducted among Gynaecologic Cancer Intergroup (GCIG) members revealed that some of the fertility preservation procedures were offered by all centres, while 20.3% offered ART, 47.3% VRT and 58.1% ST [21]. The differences in indications were based mainly on local preferences and experiences rather than based on certain criteria. It was seen that European centres preferred cone biopsy and vaginal trachelectomy compared to the USA or Japanese centres that perform ART more often. Looking at the procedure chosen based on the stage, cone biopsy was performed mainly in IA1 tumours, whereas simple trachelectomy in IA2 tumours and ART or VRT in IB1 smaller than 2 cm.

**Figure 1.**
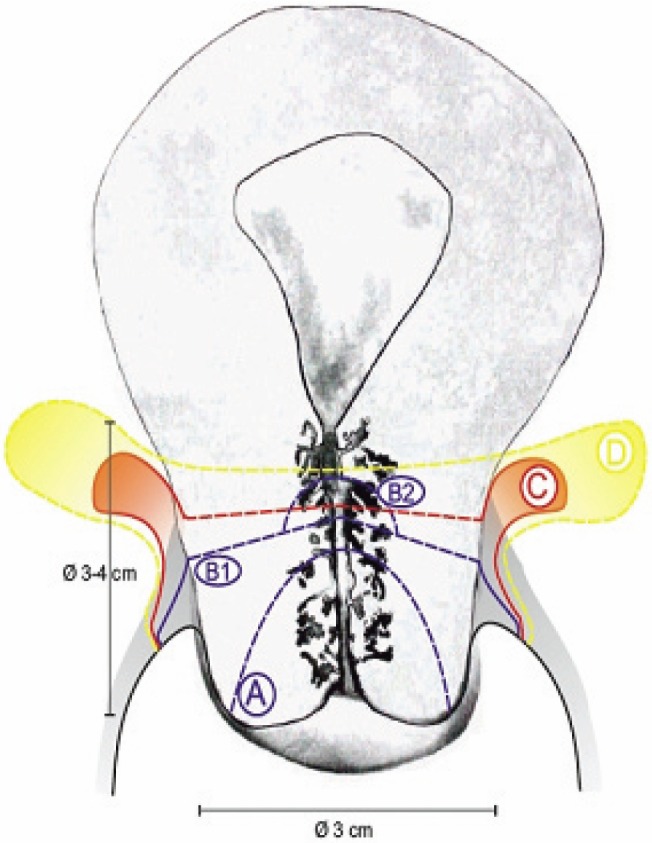
Comparison of radicality for different surgical approaches. A: extent of cone biopsy. B1: extent of simple trachelectomy. B2+C: extent of vaginal radical trachelectomy. D: extent of abdominal radical trachelectomy.

Figure 1 shows the different radicality of the described surgical techniques.

## Complications

The complication rate of VRT is comparable to laparoscopically assisted radical hysterectomy [29]. The most common peroperative complication is an injury to the urinary tract. Postoperative complications include dysmenorrhea in 24%, dyspareunia in 20% and menstrual abnormalities in 17% [30]. ART follows similar steps as in open radical hysterectomy and shows a comparable complication rate. Inflammatory complications occur in 8.6% of the patients [6]. A typical fertility-preserving complication is a stenosis of the cervical channel, which occurs in approximately 9–10% of the patients regardless of whether the approach is vaginal or abdominal [6, 31].

## Other possibilities

Detection of SLNs using SLNM is a concept that has been verified for several malignancies, including breast and vulvar cancer. In cervical cancer, the procedure is still under investigation and thus validation of this technique is ongoing. Meanwhile, we can use the technique in the early stage cervical cancer to determine the most important lymph node/lymph nodes that drain the cervix of the uterus, and therefore, the advantage is the precision of pathologic examination. Another advantage of SLN detection is to determine patients for fertility-sparing procedures already during the first procedure in order to convert patients with positive SLNs into a radical treatment group.

### Minimally invasive surgery

Other modifications of original surgical techniques have been described elsewhere. A Korean series on 79 patients describes a laparoscopic radical trachelectomy (LRT) with promising results (radicality similar to ART and an acceptable mean operating time of 291 min) [32]. Robotic surgery has been also incorporated into fertility-sparing techniques. The operating time seems comparable to open procedures with less blood loss and shorter hospital length of stay [33].

### Neoadjuvant chemotherapy

Patients with tumours greater than 2 cm in diameter represent a group of patients which is due to high rate of nodal involvement excluded from fertility preserving attempts. Use of a neoadjuvant chemotherapy has been presented by several groups as a means to downstage the disease and allow the application of a fertility-preserving surgical procedure. In patients with tumours up to 3 cm in size Maneo and co-authors used a combination of cisplatine, paclitaxel and ifosfamide (TIP) in spinocellular carcinomas and cisplatine, paclitaxel with doxorubicin (TEP) in adenocarcinomas every 3 weeks followed by cold knife conisation and lymphadenectomy [34]. No recurrence of the disease has been seen in their set of 21 patients with 6/16 pregnancies. In patients with IB1 over 2 cm and IB2 patients Robova *et al* applied a dose-dense regime using cisplatine with ifosfamide in spinocellular cancers and cisplatine with doxorubicin in adenocarcinomas every 10 days [35]. Chemotherapy was followed by laparoscopic lymphadenectomy and ST. In this series, 10/20 patients became pregnant resulting in eight deliveries. A small series has been published by Plante *et al* using TIP/TEP regimen [36].

## Obstetrical outcomes and care after trachelectomy

Over 900 cases of trachelectomy have been reported in the literature. The overall pregnancy rate is 30% for VRT and 15% for ART [30]. For ST, the pregnancy rate is about 50%. However, the number of patients is still low though rapidly increasing [37]. It is clearly seen that the larger the damage to the paracervical tissue caused by the surgery, the lower the chance of conception. Premature labour before the 32nd week and between the 32nd and 37th week of pregnancy has been observed in approximately 12% and 28% in VRT and ART, respectively [30]. The shortening of the cervix plays an important role in the risk of premature delivery here as proven in patients after cone biopsy (RR about 2) [38].

An integral part of fertility-preserving procedures is encouragement and close counselling with the patient before and throughout pregnancy. Preferably, the ideal arrangement is to follow the patient during pregnancy at the same department in which the surgery was performed. There is no consensus among authors about the interval between surgery and the first attempt to conceive, but a minimum of 3 months seems to be justified.

Another issue in regards to postoperative care concerns prophylactic treatment during pregnancy. Our department administers cephalosporin antibiotics at week 16, 20 and 24 and clindamycin vaginal treatment to prevent intraovulary infection at week 16 and 24. Any suspicion of premature labour should be carefully examined and collaboration with a department equipped with a neonatal intensive care unit is obligatory. Other authors prefer prophylactic use of oral metronidazol during week 15–21 and sexual abstinence during the 2nd and 3rd trimester [39].

An issue of preventive cerclage is an unresolved question. Some centres prefer to place a preventative cerclage during oncological surgery [40, 41], whereas other centres prefer that placement to be done during pregnancy [42].

## Conclusions

Currently, in selected sets of patients (tumours smaller than 2 cm without parametrial and nodal involvement) VRT combined with laparoscopic lymphadenectomy is the standard fertility-preserving procedure for treatment. Oncological safety has been confirmed for VRT as well as for ART. Promising results have been reported for ST in terms of oncological results. Positive pregnancy outcomes have been reported in ascending order of ART, VRT and ST. Use of neoadjuvant chemotherapy is an experimental modality for patients who do not fulfil initial indication criteria.

## Conflicts of interest

All the authors declare no conflict of interest.

## References

[1] Ferlay J (2013). http://globocan.iarc.fr.

[2] Robustillo SA (2013). EU employment and social situation. Quarterly Review.

[3] Dargent D, Burn JL, Roy M (1994). La trachélectomie élargie (TE). Une alternative á l´hystérectomie radicale dans le traitement des cancers infiltrans développés sur la face externe du col utérin. J Obstet Gynecol.

[4] Smith JR (1997). Abdominal radical trachelectomy: a new surgical technique for the conservative management of cervical carcinoma. Br J Obstet Gynaecol.

[5] Beiner ME (2008). Radical vaginal trachelectomy vs radical hysterectomy for small early stage cervical cancer: a matched case-control study. Gynecol Oncol.

[6] Pareja R (2013). Surgical, oncological, and obstetrical outcomes after abdominal radical trachelectomy - a systematic literature review. Gynecol Oncol.

[7] Covens A (2002). How important is removal of the parametrium at surgery for carcinoma of the cervix?. Gynecol Oncol.

[8] Kinney WK (1995). Identification of a low-risk subset of patients with stage IB invasive squamous cancer of the cervix possibly suited to less radical surgical treatment. Gynecol Oncol.

[9] Rob L (2005). Study of lymphatic mapping and sentinel node identification in early stage cervical cancer. Gynecol Oncol.

[10] Steed H (2006). Early cervical cancer and parametrial involvement: is it significant?. Gynecol Oncol.

[11] Stegeman M (2007). The incidence of parametrial tumor involvement in select patients with early cervix cancer is too low to justify parametrectomy. Gynecol Oncol.

[12] Rob L (2008). A less radical treatment option to the fertility-sparing radical trachelectomy in patients with stage I cervical cancer. Gynecol Oncol.

[13] Lintner B (2013). Use of abdominal radical trachelectomy to treat cervical cancer greater than 2 cm in diameter. Int J Gynecol Cancer.

[14] Wethington SL (2013). Expanding the indications for radical trachelectomy: a report on 29 patients with stage IB1 tumors measuring 2 to 4 centimeters. Int J Gynecol Cancer.

[15] Li J (2013). Abdominal radical trachelectomy: Is it safe for IB1 cervical cancer with tumors >/= 2 cm?. Gynecol Oncol.

[16] Fischerova D (2008). Transrectal ultrasound and magnetic resonance imaging in staging of early cervical cancer. Int J Gynecol Cancer.

[17] Selman TJ (2008). Diagnostic accuracy of tests for lymph node status in primary cervical cancer: a systematic review and meta-analysis. CMAJ.

[18] Signorelli M (2011). Preoperative staging of cervical cancer: is 18-FDG-PET/CT really effective in patients with early stage disease?. Gynecol Oncol.

[19] Rob L (2012). Surgical options in early cervical cancer. Int J Hyperthermia.

[20] Lukas R (2013). Current status of sentinel lymph node mapping in the management of cervical cancer. Expert Rev Anticancer Ther.

[21] Lindsay R (2012). Survey on the management of early cervical cancer among members of the GCIG. Int J Gynecol Cancer.

[22] Landoni F (2007). Chemo-conization in early cervical cancer. Gynecol Oncol.

[23] Shepherd JH, Milliken DA (2008). Conservative surgery for carcinoma of the cervix. Clin Oncol (R Coll Radiol).

[24] Park KJ (2008). Frozen-section evaluation of cervical adenocarcinoma at time of radical trachelectomy: pathologic pitfalls and the application of an objective scoring system. Gynecol Oncol.

[25] Wan XP (2006). Abdominal radical trachelectomy: two new surgical techniques for the conservation of uterine arteries. Int J Gynecol Cancer.

[26] Cibula D (2009). Abdominal radical trachelectomy in fertility-sparing treatment of early-stage cervical cancer. Int J Gynecol Cancer.

[27] Rob L (2007). Less radical fertility-sparing surgery than radical trachelectomy in early cervical cancer. Int J Gynecol Cancer.

[28] Plante M (2013). Simple vaginal trachelectomy in early-stage low-risk cervical cancer: a pilot study of 16 cases and review of the literature. Int J Gynecol Cancer.

[29] Marchiole P (2007). Oncological safety of laparoscopic-assisted vaginal radical trachelectomy (LARVT or Dargent’s operation): a comparative study with laparoscopic-assisted vaginal radical hysterectomy (LARVH). Gynecol Oncol.

[30] Schneider A (2012). Clinical recommendation radical trachelectomy for fertility preservation in patients with early-stage cervical cancer. Int J Gynecol Cancer.

[31] Hertel H (2006). Radical vaginal trachelectomy (RVT) combined with laparoscopic pelvic lymphadenectomy: prospective multicenter study of 100 patients with early cervical cancer. Gynecol Oncol.

[32] Park JY (2014). Long-term outcomes after fertility-sparing laparoscopic radical trachelectomy in young women with early-stage cervical cancer: An Asan Gynecologic Cancer Group (AGCG) study. J Surg Oncol.

[33] Nick AM (2012). Fertility sparing surgery for treatment of early-stage cervical cancer: open vs robotic radical trachelectomy. Gynecol Oncol.

[34] Maneo A, Chiari S, Bonazzi C, Mangioni C (2008). Neoadjuvant chemotherapy and conservative surgery for stage IB1 cervical cancer. Gynecol Oncol.

[35] Robova H (2014). Oncological and pregnancy outcomes after high-dose density neoadjuvant chemotherapy and fertility-sparing surgery in cervical cancer. Gynecol Oncol.

[36] Plante M (2006). Neoadjuvant chemotherapy followed by vaginal radical trachelectomy in bulky stage IB1 cervical cancer: case report. Gynecol Oncol.

[37] Rob L, Skapa P, Robova H (2011). Fertility-sparing surgery in patients with cervical cancer. Lancet Oncol.

[38] Bruinsma FJ, Quinn MA (2011). The risk of preterm birth following treatment for precancerous changes in the cervix: a systematic review and meta-analysis. BJOG.

[39] Persson J (2012). Reproducibility and accuracy of robot-assisted laparoscopic fertility sparing radical trachelectomy. Gynecol Oncol.

[40] Abu-Rustum NR (2008). Surgical and pathologic outcomes of fertility-sparing radical abdominal trachelectomy for FIGO stage IB1 cervical cancer. Gynecol Oncol.

[41] Nishio H (2009). Abdominal radical trachelectomy as a fertility-sparing procedure in women with early-stage cervical cancer in a series of 61 women. Gynecol Oncol.

[42] Pareja FR (2008). Abdominal radical trachelectomy for invasive cervical cancer: a case series and literature review. Gynecol Oncol.

